# Ionizing radiation and inhibition of angiogenesis in a spontaneous mammary carcinoma and in a syngenic heterotopic allograft tumor model: a comparative study

**DOI:** 10.1186/1748-717X-6-66

**Published:** 2011-06-08

**Authors:** Oliver Riesterer, Christoph Oehler-Jänne, Wolfram Jochum, Angela Broggini-Tenzer, Van Vuong, Martin Pruschy

**Affiliations:** 1 Dept. Radiation Oncology, University Hospital Zurich, CH-8091 Zurich; 2 Dept. Pathology, University Hospital Zurich, CH-8091 Zurich; 3 Institute of Pathology, Kantonsspital St. Gallen, CH-9007 St.Gallen

## Abstract

**Background:**

The combined treatment modality of ionizing radiation (IR) with inhibitors of angiogenesis (IoA) is a promising treatment modality based on preclinical *in vivo *studies using heterotopic xeno- and allograft tumor models. Nevertheless reservations still exist to translate this combined treatment modality into clinical trials, and more advanced, spontaneous orthotopic tumor models are required for validation to study the efficacy and safety of this treatment modality.

**Findings:**

We therefore investigated the combined treatment modality of IR in combination with the clinically relevant VEGF receptor (VEGFR) tyrosine kinase inhibitor PTK787 in the MMTV/c-neu induced mammary carcinoma model and a syngenic allograft tumor model using athymic nude mice. Mice were treated with fractionated IR, the VEGFR-inhibitor PTK787/ZK222584 (PTK787), or in combination, and efficacy and mechanistic-related endpoints were probed in both tumor models. Overall the treatment response to the IoA was comparable in both tumor models, demonstrating minimal tumor growth delay in response to PTK787 and PTK787-induced tumor hypoxia. Interestingly spontaneously growing tumors were more radiosensitive than the allograft tumors. More important combined treatment of irradiation with PTK787 resulted in a supraadditive tumor response in both tumor models with a comparable enhancement factor, namely 1.5 and 1.4 in the allograft and in the spontaneous tumor model, respectively.

**Conclusions:**

These results demonstrate that IR in combination with VEGF-receptor tyrosine kinase inhibitors is a valid, promising treatment modality, and that the treatment responses in spontaneous mammary carcinomas and syngenic allografts tumor models are comparable.

## Findings

Preclinical studies have demonstrated that the combined treatment of IR with IoA is highly effective in xeno- and allograft tumor models of breast cancer [1-3]. It is generally agreed that IR and IoA interact on the level of the tumor microenvironment, although the exact mechanism of synergistic action of these two treatment modalities is still a matter of debate. For example, IoAs can either improve tumor oxygenation by a mechanism termed vascular normalization [4] and thereby sensitize for IR, or IoAs can increase tumor hypoxia [3,5-7], which is counteracted by combined treatment with IR. The cause for these different treatment responses to IoAs is unknown but might be related to differences in the mode of action of the IoAs and the treatment regimens including doses and scheduling, and the tumor models used on the preclinical level [8]. With respect to the tumor models used on the preclinical level, most studies were performed with either orthotopic or heterotopic xenograft [4-6] or heterotopic allograft tumor models [3]. Though, little is known about the relevance of a differential microenvironment in xenograft versus allograft and heterotopic versus orthotopic tumors with regard to the treatment response to IoAs and in particular to a combined treatment modality of IoAs with IR [9,10]

We previously demonstrated that the risk of enhanced tumor hypoxia in response to the inhibitor of vascular endothelial growth factor receptor 2 (VEGFR2) PTK787/ZK222584 (PTK787) exists, but is minimal when PTK787 is combined with IR [3]. Our experiments were originally performed in a classic allograft tumor model derived from NF9006 tumor cells, which were originally established from spontaneous murine MMTV/c-neu driven mammary carcinomas. These fast-growing allografts and their fast-developing tumor vasculature might not represent the tumor microenvironment in a spontaneously growing tumor. We therefore revisited the potential drawback of this artificial fast-growing tumor model in the corresponding MMTV/(c-neu)-driven spontaneously growing mammary tumor model and compared the treatment-dependent responses with those achieved in the syngenic allografts.

The female, heterozygous offspring of female FVB-wild type mice, which were mated with male FVB-Tg(MMTV/c-neu) mice (Charles River), developed mammary carcinomas within 100 days after a first littering. To generate the corresponding allograft tumor model, mammary carcinoma cells (NF9006), which were established from the spontaneous tumor model, were subcutaneously injected (4 × 10^6 ^cells) on the back of athymic nude mice. Spontaneous tumors and allografts were allowed to grow to 200 mm^3 ^± 10% before start of treatment. Mice carrying allograft tumors on their backs were irradiated using a customized shielding device whereas mice with spontaneous tumors in the mouse breast were given upper-half-body radiotherapy. All mice were treated with a minimally fractionated locoregional radiotherapy regimen of 4 × 3 Gy during 4 consecutive days, using a Pantak Therapax 300-kV X-ray unit at 0.7 Gy/min. PTK787 (dissolved in 5% DMSO, 1% Tween-80 and 94% H_2_O) was applied orally either alone (100 mg/kg) or 1 hour prior to irradiation. Immunohistochemical stainings of tumor sections were performed after tumor excision at day 4 of treatment. Detailed descriptions of the experimental procedures are given elsewhere [11]. The Student's t-test was used to statistically analyse the differences between the treatment groups.

**Figure 1:**
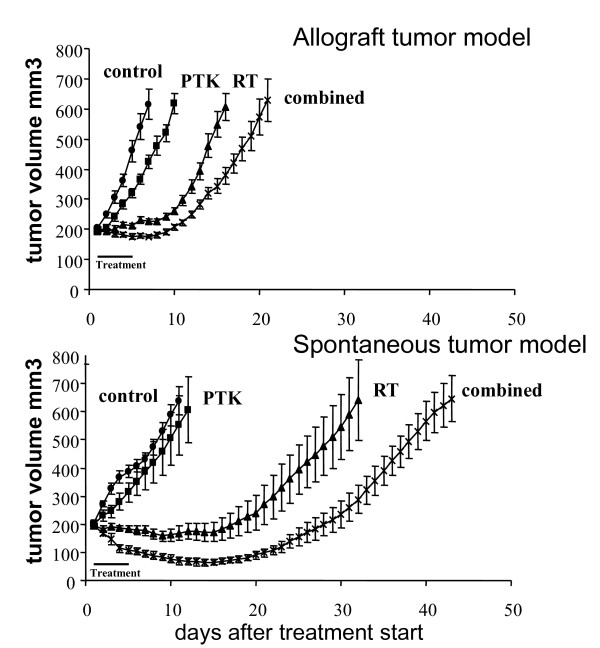
**Similar treatment response in spontaneous mammary carcinoma and in a syngenic heterotopic allograft tumor model**. Tumor growth delay of syngenic mammary carcinoma allografts (A) and orthotopic spontaneous mammary carcinomas (B) in response to IR (4 × 3 Gy) and PTK787 (4 × 100 mg/kg) alone and in combination. For the allograft tumor model 10-15 mice/group and for the spontaneous tumor model 8-13 mice/group were used. Each curve represents the mean tumor volume per group ± SE.

In comparison to the fast growing allograft tumor model, which formed tumors in average within 14 days after cell injection, orthotopic heterozygous FVB-Tg(MMTV/c-neu) tumor formation required more than 100 days to reach the minimal treatment size of 200 mm^3^. Thereafter tumor growth rates were comparable between the two tumor models. Absolute tumor growth delay (AGD) in response to treatment with the IoA PTK787 alone was minimal in both tumor models (Figure 1).

**Figure 2:**
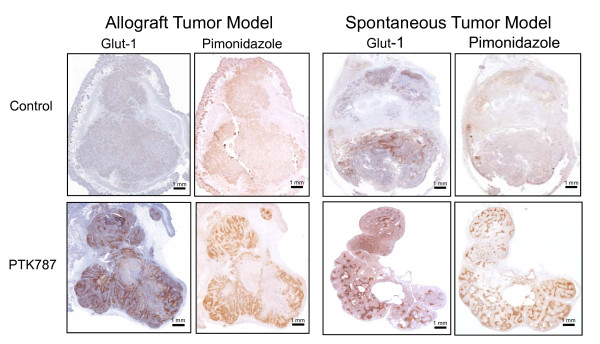
**Increased tumor hypoxia in response to PTK787-treatment**. Immunohistochemical detection of tumor hypoxia with antibodies against endogenous Glut-1 or the exogenous 2-nitroimidazole hypoxia marker pimonidazole hydrochloride in NF9006-derived allografts and spontaneous mammary carcinomas. Mice with NF9006-derived allografts and spontaneous tumors were treated with PTK787 (100 mg/kg × 4). Mice were sacrificed and tumors were harvested on day 4 of treatment.

We previously observed a PTK787-induced increase of tumor hypoxia using Glut-1 and pimonidazole staining [3,11]. The hypoxia probe pimonidazole, which specifically accumulates in hypoxic tissue areas, was injected 45 min before mice killing. Two adjacent tumor sections were then probed either with antibodies specific for the endogenous hypoxia marker Glut-1 or for pimonidazole. A speckled strongly-increased staining pattern with both hypoxia markers was observed in response to PTK787-treatment in both allografts and spontaneous tumors, demonstrating a similar treatment response to this IoA on the level of tumor hypoxia (Figure 2).

Interestingly, spontaneously growing tumors were much more sensitive to treatment with IR alone, with an AGD of 20.1 days to triple the initial treatment volume in comparison to an AGD of 8.4 days for allograft tumors (P < 0.001). Combined treatment with PTK787 and IR resulted in a supra-additive treatment response in both tumor models with an AGD of 14 and 30.4 days in the allograft and the spontaneous tumor model, respectively (Figure 1, Table 1). More important the enhancement factor was comparable for the two tumor models, namely 1.5 and 1.4 in the allograft and in the spontaneous tumor model, respectively.

**Table 1:** Results of Growth Delay Assays

Schedule	**Time in days for tumors to grow from 200 to 600 mm**^**3**^	**Growth-AGD**^**††**^	**Delay NGD**^**§**^	**Endhancement Factor**^**ǁ**^
*Allografts*				
Control	8.8 ± 0.9	-		
PTK787	10.5 ± 0.5	1.7	-	-
IR	17.2 ± 0.5	8.4	-	-
PTK787+IR	22.8 ± 1.4	14.0	12.3	1.5
*Spontanous Tumors*				
Control	11.5 ± 0.8	-	-	-
PTK787	14.3 ± 1.5	2.8	-	-
IR	31.6 ± 2.8	20.1	-	
PTK787+IR	41.9 ± 1.8	30.4	27.6	1.4

Effect of PTK787 on the radioresponse of allograft and spontaenous tumors measured by tumor growth delay. For treatment schedules see Figure 1.

††Absolute tumor growth delay (AGD) caused by PTK787, IR, or their combination is defined as the time in days required for tumor to triple tumor size from 200 to 600 mm^3 ^minus the time in days in untreated tumors.

§Normalized tumor growth delay (NGD) is defined as the time in days for tumors to triple tumor size in the mice treated with the combination of PTK787 and IR minus the time in days to triple tumor size in mice treated with PTK787 alone.

ǁEnhancement factors obtained by dividing NGD in mice treated with PTK787 plus IR by the AGD in mice treated with IR alone.

**Figure 3:**
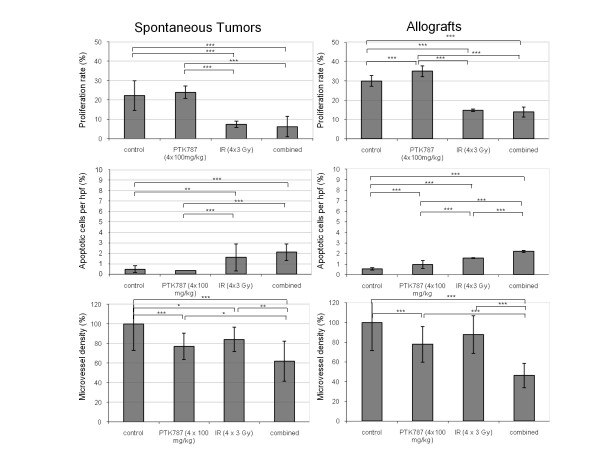
**Tumor cell proliferation, apoptosis, and microvessel density in response to PTK787 and IR**. Mice with NF9006-derived allografts or spontaneous mammary carcinomas were treated with PTK787 (100 mg/kg × 4), IR (4 Gy × 3), or in combination. At day 4 of treatment, mice were sacrificed, and tumors were harvested, formalin fixed, and stained for the Ki-67 (A), TUNEL (B), and CD31 (C) as marker of tumor cell proliferation, apoptosis, and microvessel density, respectively. Percentage CD31-positive cells and Ki-67- and TUNEL-positive nuclei per high-powered fields (hpf) was determined in 4-10 randomly chosen visual fields in each of at least two similarly treated vital tumor tissues of allografts and orthotopic tumors. For the allograft tumor tissue sections 3 mice/group and for the spontaneous tumor model tissue sections 2-4 mice/group were used. Each bar represents the mean value per group ± SD (*<0.05; **<0.01; ***<0.005).

Tumor cell apoptosis and tumor cell proliferation were investigated to analyze the effects of the two treatment modalities (Figure 3). Tumor cell proliferation was determined using immunohistochemistry for the Ki-67 protein, which is expressed during all phases of the cell cycle, except G0. In both tumors models treatment with PTK787 alone did not reduce the proliferative activity of tumor cells whereas treatment with IR significantly reduced tumor cell proliferation in comparison to control tumors (p < 0.001). Combined treatment with IR and PTK787 did not further reduce the proliferative activity in both tumor models. Tumor cell apoptosis was determined by terminal deoxynucleotidyl transferase-mediated nick-end labeling (TUNEL). Treatment with PTK787 did not induce tumor cell apoptosis in contrast to treatment with IR alone, which resulted in an approximately 3 fold increase of TUNEL-positive cells in both tumor models (allograft: p < 0.001, spontaneous: p < 0.01). Combined treatment with IR and PTK787 resulted in a similar absolute increase (appr. 5 fold) of tumor cell apoptosis in comparison to control tumors (p < 0.001). In comparison to the apoptotic treatment response to IR alone, the amount of apoptotic tumor cells in response to the combined treatment modality was significantly further enhanced, but only for allografts (p < 0.001) and not for spontaneous tumors (p = 0.3), which showed a more heterogenous staining pattern. Nevertheless, similar treatment-dependent effects could be determined in the allograft and spontaneous tumor models on the tumor cell level. To examine a change in microvessel density in response to the different treatment modalities, αCD31-stained tumor vessels were counted in histological sections. The microvessel density (MVD) was reduced on treatment with PTK787 alone (p < 0.01) and was further decreased on treatment with PTK787 and IR in combination (allograft p < 0.001, spontaneous p < 0.05), again to a similar extent in both tumor types (Figure 3). Thus, on the level of the tumor microenvironment a significant combined treatment effect could be observed on the level of the tumor vasculature. Of note the spontaneous mammary carcinoma tissue contained large lake-like cavities or vessels but the MVD was similar to the allograft tissue (see above Figure 2).

Here, we have examined the effects of the combined treatment modality of ionizing radiation with the VEGF-receptor tyrosine kinase inhibitor PTK787 in both a spontaneous and a strongly related allograft mammary carcinoma model. Little is known about differences in the make-up of the tumor microenvironment between allografts and xenografts, orthotopic and heterotopic tumors. In the models used in this study, major differences with regard to the tumor biology, and eventually to the treatment response, would rather be expected on the level of the tumor microenvironment than on the level of the syngenic tumor cells. Interestingly we observed the strongest difference between the two tumor models on the level of radiation sensitivity. The NF9006 cell line, which is derived from a spontaneous murine MMTV/c-neu driven mammary carcinoma, may have acquainted additional mutations during the *in vitro *establishment, and these genetic mutations might contribute to the increased radiation resistant phenotype of allografts derived from this cell line. On the other hand, increased radiation sensitivity of spontaneous tumors in comparison to allograft tumors may be linked to differences in the tumor vasculature as well as immunomodulatory effects in the immunocompetent host [12]. PTK787 exerts its antivascular effects by targeting the VEGF receptor, which is almost exclusively located on endothelial cells. The treatment responses to PTK787 alone were similar in both tumor models, which indicate a similar phenotype and treatment sensitivity of the respective endothelial cells. This is further supported by a similar treatment-dependent reduction of microvessel densities and a treatment-dependent increase of tumor hypoxia.

We previously demonstrated that IoA induce tumor hypoxia in allografts, which is counteracted by combined treatment with irradiation [3]. Eventually combined treatment results in a supraadditive treatment response. Insofar our studies are of high clinical interest since PTK787 exerted a similar treatment response in the allograft and the spontaneously growing tumor model with potent radiation enhancement to a similar extent in both tumor models. Thereby the data strengthen the evidence to overcome a major obstacle translating such a treatment combination into the clinics, i.e. the supposition that a potential IoA-dependent increase of tumor hypoxia might impair the treatment response to ionizing radiation. Obtaining preclinical data with spontaneous tumor models is highly laborious and cost-effective. Our comparative study furthermore demonstrates that an allograft tumor model is adequate and represents a valid tumor model to investigate the combined treatment modality of IR with IoA.

## Competing interests

The authors declare that they have no competing interests.

## Authors' contributions

OR and CO-J carried out the in vivo studies and drafted the manuscript. WJ carried out the immunohistochemistry experiments. AB-T and VV participated in the in vivo studies. MP conceived of the study, participated in its design and coordination and finalized the manuscript. All authors read and approved the final manuscript.
